# Total Intravenous Anaesthesia with High-Dose Remifentanil Does Not Aggravate Postoperative Nausea and Vomiting and Pain, Compared with Low-Dose Remifentanil: A Double-Blind and Randomized Trial

**DOI:** 10.1155/2014/724753

**Published:** 2014-06-03

**Authors:** Seong-Hyop Kim, Chung-Sik Oh, Tae-Gyoon Yoon, Min Jeng Cho, Jung-Hyun Yang, Hye Ran Yi

**Affiliations:** ^1^Department of Anaesthesiology and Pain Medicine, Konkuk University Medical Center, Konkuk University School of Medicine, Seoul, Republic of Korea; ^2^Institute of Biomedical Science and Technology, Konkuk University School of Medicine, Seoul, Republic of Korea; ^3^Department of Surgery, Konkuk University Medical Center, Konkuk University School of Medicine, Seoul, Republic of Korea; ^4^Post-Anaesthetic Care Unit, Konkuk University Medical Center, Konkuk University School of Medicine, Seoul, Republic of Korea

## Abstract

The study was designed to investigate postoperative nausea and vomiting (PONV) in low- and high-dose remifentanil regimens for total intravenous anaesthesia (TIVA) in adult female patients with American Society of Anaesthesiologists physical status classification I undergoing local breast excision. Propofol and remifentanil 5 ng*·*mL^−1^ (L group) or 10 ng*·*mL^−1^ (H group) were administered for anaesthesia induction and maintenance. Propofol was titrated within range of 0.1 **μ**g*·*mL^−1^ to maintain bispectral index (BIS) values between 40 and 60. Haemodynamic parameters during the intra- and postoperative periods and 24 h postoperative visual analogue scale (VAS) and PONV were evaluated. Each group with 63 patients was analyzed. The H group showed higher use of remifentanil and lower use of propofol, with similar recovery time. Mean systemic arterial blood pressure (MBP), heart rate, and BIS did not differ significantly before and after endotracheal intubation in the H group. However, significant increases in MBP and BIS were apparent in the L group. Postoperative VAS, PONV incidence and scale, and Rhodes index did not differ significantly between the two groups. In conclusion, TIVA with high-dose remifentanil did not aggravate PONV with similar postoperative pain, compared with low-dose remifentanil. Furthermore, high-dose remifentanil showed more haemodynamic stability after endotracheal intubation. This trial is registered with KCT0000185.

## 1. Introduction

Postoperative nausea and vomiting (PONV) is a major concern in patients undergoing general anaesthesia and may increase patient discomfort, delay discharge, and increase costs of care. Inhalational anaesthetic agents may be one of the many contributors to emesis [1]. Total intravenous anaesthesia (TIVA) is preferred to avoid PONV for patients undergoing general anaesthesia [2]. For improved haemodynamic and surgical states, the combination of propofol as a hypnotic agent and remifentanil as an analgesic agent is the most popular TIVA regimen [3–5].

Remifentanil is an esterase metabolized opioid with a rapid clearance, which is widely used in general anaesthesia, especially for outpatients [6]. As a result of rapid clearance, it is expected that remifentanil is associated with less PONV, even though opioid use is a risk factor of PONV. However, PONV with remifentanil has been inconsistent in clinical situations. Rama-Maceiras et al. reported that propofol with remifentanil had a lower incidence of PONV and requirement for antiemetic drugs in patients undergoing plastic surgery, compared with fentanyl [7]. Mukherjee et al. reported that patients with TIVA using propofol and remifentanil experienced significantly less PONV with a reduced requirement for antiemetics, compared with balanced anaesthesia using propofol, isoflurane, and fentanyl in middle ear surgery, even though the initial pain score was higher in patients with TIVA using propofol and remifentanil [8]. On the contrary, Del Gaudio et al. reported no difference of PONV between remifentanil and fentanyl in target-controlled infusion (TCI) of propofol for elective supratentorial craniotomy [9]. Morino et al. identified remifentanil use during surgery as a risk factor for PONV [10]. Gaszynski et al. reported that morbidly obese patients receiving remifentanil had higher rates of PONV and postoperative pain, compared with patients treated with fentanyl or alfentanil during open Roux-en-y gastric bypass [11]. Additionally, there is a paucity of literature regarding PONV with different doses of remifentanil.

The study was designed to investigate PONV in low- and high-dose remifentanil regimens for TIVA in adult female patients with American Society of Anaesthesiologists physical status (ASA PS) classification I undergoing local breast excision. The intraoperative haemodynamic parameters and postoperative pain were also evaluated.

## 2. Materials and Methods

### 2.1. Study Population

The study was approved by the Institutional Review Board of Konkuk University Medical Center, Seoul, Korea (KUH1160020), and registered at http://cris.nih.go.kr/ (KCT0000185). Written informed consent was obtained from each patient and the study was conducted in a prospective, double-blind, and randomized fashion. Adult female patients with ASA PS classification I undergoing local excision of breast under admission were enrolled. Patients were excluded if the following criteria were present: (1) patient age < 20 years, (2) redo case, (3) concurrent other surgery, (4) allergy to egg or soybean oil, (5) history of drug abuse, (6) receiving current medications, and (7) demand of patient-controlled analgesia (PCA). The patients admitted at a day before the operation. The written informed consent for the study and the preoperative interview with anaesthesia permission were obtained from the investigators and the anaesthesiologists who were expected to participate in patient care and blind to the study, respectively, at the same day. They were delivered at the day of the operation when the patient was transferred to the reception room for the operation. And then, the registered nurse (RN) who did not participate in patient care and was blind to the study performed all randomization processes. The patients were allocated randomly to receive either propofol-low dose remifentanil (L group) or propofol-high dose remifentanil (H group) for TIVA through the random assignment, performed by the RN, using sealed envelopes with the options inside L or H before anaesthesia induction. The equal numbers of sealed envelopes with the options with L for L group and H for H group were included in a sealed envelope. When a patient was dropped out of the study, the sealed envelope with the same option for the patient was added into the sealed envelope. The randomization was ended when the sealed envelopes with the options inside L and H were run out. The RN also prepared the TCI devices for the study before anaesthesia induction.

All data were collected by trained observers who did not participate in patient care and were blinded to the study.

### 2.2. Anaesthetic Technique

The anaesthetic technique was standardized. The patient arrived at the operation room without premedication. After establishing routine systemic blood pressure monitoring and noninvasive patient monitoring (pulse oximetry, electrocardiography, and bispectral index (BIS)), anaesthesia was induced. The anaesthesiologists who participated in patient care but were blinded to the study were requested to anaesthetize the patients as described below. Lidocaine 0.5 mg·kg^−1^ was administered to decrease pain induced by propofol. An initial target concentration (effect-site, modified Marsh model with a *k*_*e*0_ of 1.21 min^−1^ [12]) of propofol 4 *μ*g·mL^−1^ and the fixed target concentration (plasma site, Minto model [13, 14]) of remifentanil 5 ng·mL^−1^ (L group) or 10 ng·mL^−1^ (H group) were administered using two TCI devices. The target concentrations of remifentanil in the L and H groups were achieved by 10 min of administration and maintained during anaesthesia. The fixed target concentration of remifentanil, prepared with TCI according to randomization by the RN who participated in the patients' allocation for the study, was blinded to the anaesthesiologists by sealing the monitor of the TCI device. An initial target concentration of propofol was titrated more or less than 0.1 *μ*g·mL^−1^ to maintain BIS values between 40 and 60. Rocuronium 0.6 mg·kg^−1^ was administered for muscle relaxation after loss of consciousness under the guidance of peripheral neuromuscular transmission monitoring. Endotracheal intubation was performed after 10 min from the start of operation of the TCI device for remifentanil with a train-of-four count of 0. After the induction of anaesthesia, patients were ventilated with 40% oxygen in air. The tidal volume was 6 mL·kg^−1^ of ideal body weight and positive end-expiratory pressure was not utilized. The respiratory rate was adjusted to keep the partial pressure of end-tidal carbon dioxide between 35 and 40 mmHg. Additional rocuronium was administered under the guidance of peripheral monitoring of neuromuscular transmission. Phenylephrine 30 *μ*g (if mean systemic arterial blood pressure (MBP) was below 60 mmHg and heart rate (HR) was above 40 beats·min^−1^), ephedrine 4 mg (if MBP was below 60 mmHg and HR was below 40 beats·min^−1^), or atropine (if HR was below 40 beats·min^−1^) was injected to prevent hypotension or bradycardia. Phenylephrine was continuously infused if MBP was below 60 mmHg and was continued with repetitive phenylephrine injections. Nicardipine 0.5 mg was injected at systolic systemic BP above 180 mmHg or diastolic systemic blood pressure above 110 mmHg, and esmolol 30 mg was injected at MBP above 60 mmHg and HR above 110 beats·min^−1^ during anaesthesia after the target concentration of remifentanil 5 ng·mL^−1^ (L group) or 10 ng·mL^−1^ (H group) was achieved. TCIs of remifentanil and propofol were stopped, and ketorolac 0.5 mg·kg^−1^ was injected intravenously for postoperative pain control at the end of the surgery. Residual neuromuscular paralysis was antagonized with neostigmine 0.05 mg·kg^−1^ and glycopyrrolate 0.01 mg·kg^−1^ under the guidance of peripheral neuromuscular transmission monitoring. After endotracheal extubation, patient was transferred to postanaesthetic care unit (PACU).

### 2.3. Measurement

At arrival at the operation room, MBP, HR, and BIS were measured as baseline values (T0) just before endotracheal intubation (T1), just after endotracheal intubation (T2), and on arrival at PACU (T3). In patients receiving phenylephrine, ephedrine, or atropine, total dose of phenylephrine, ephedrine, atropine, nicardipine, or esmolol was recorded.

Postoperative pain and PONV were evaluated by RN who was blinded to the study at the PACU and general ward. Pain was assessed using a visual analogue scale (VAS) ranging from 0 to 100 with 0 being no pain and 100 being the worst pain imaginable on arrival at the PACU (T3), 30 minutes after arrival at PACU (T4), 6 h after discharge from the PACU (T5), and 24 h after discharge from PACU (T6). Ketorolac 0.5 mg·kg^−1^ was administered intravenously on demand at the PACU for postoperative analgesia and recorded. PONV was assessed using a three-point ordinal scale (0 = none, 1 = nausea, 2 = retching, and 3 = vomiting) [15] at the same points. Nausea was defined as a subjectively unpleasant sensation associated with awareness of the urge to vomit. Retching was defined as the labored, spasmodic, and rhythmic contraction of the respiratory muscles without expulsion of gastric contents. Vomiting was defined as the forceful expulsion of gastric contents from the mouth. The existence of PONV was defined as nausea, retching, or vomiting. The severity of PONV from T4 to T5 and from T5 to T6 was evaluated using a modified Rhodes index [16]. Ondansetron 0.1 mg·kg^−1^ was intravenously given for antiemetic treatment on demand and recorded.

Total doses of remifentanil and propofol were recorded with the TCI devices. Anaesthesia time (from TCI start to discharge from operation room) and operation time (from skin incision to end of surgery) were recorded. Recovery time (from TCI stop to discharge from the operation room) was also recorded.

### 2.4. Statistical Analysis

From a pilot study with 20 female patients undergoing local excision of breast under TIVA with low-dose remifentanil and propofol, PONV incidence and scale and Rhodes index at T5 were 15% (six patients), 0.30 ± 0.47 and 1.10 ± 1.89, respectively. The primary and secondary outcomes were Rhodes index at T5 and PONV incidence at T5, respectively. A minimum detected difference of double-PONV incidence, -PONV scale, and -Rhodes index between the groups was considered to be of clinical significance. The sample sizes of 63, 28, and 33 were calculated with a power of 0.9 and an *α* value of 0.05.

The data was analyzed by the statistician who was blind to the study, using the program Statistical Package for the Social Sciences ver. 11.0. The intragroup changes in MBP, HR, PONV, and VAS over time were analyzed using an analysis of variance on ranks for repeated measurements (Friedmann) and if significant, a Tukey's test was performed to compare the variables with the baseline value. The values between the L and H groups were analyzed using an unpaired Chi-square test, Fisher's exact test, or Mann-Whitney Rank Sum test. All data were expressed as the number of patients or mean ± standard deviation. A value of *P* < 0.05 was considered statistically significant.

## 3. Results

**Figure 1:**
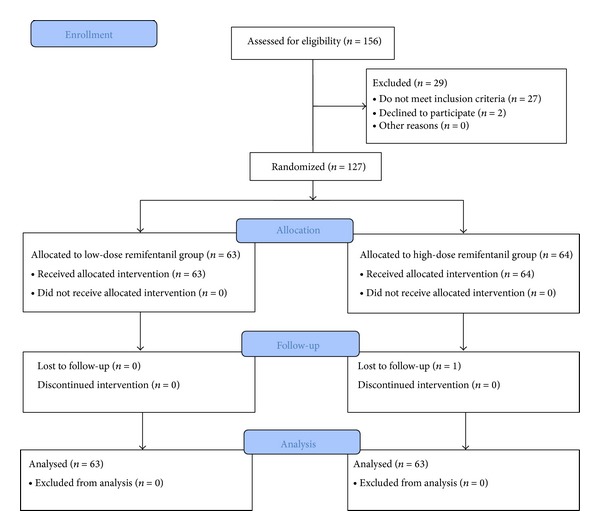
CONSORT flow diagram for the study.

One hundred and fifty-six patients were eligible for the study. Twenty-seven patients were excluded: 5 patients for redo case, 1 patient for another concurrent surgery, 1 patient for allergy to egg or soybean oil, 15 patients for receiving current medications, and 5 patients for demand of PCA. Two patients declined to participate. One patient in the H group withdrew at T6. Thus, total 126 patients with 63 patients for each group were included in the final analysis (Figure 1).

The demographic profiles were similar between the two groups. The H group showed a higher use of remifentanil (1894 ± 735 versus 1013 ± 436 *μ*g; *P* < 0.001) and lower use of propofol (428 ± 138 versus 580 ± 294 mg; *P* < 0.001), but there was no significant difference in recovery time (13 ± 4 versus 14 ± 6 min; *P* = 0.82) (Table 1).

Concerning haemodynamic changes, MBP and HR at T0 and T3 were not significantly different between the two groups. MBP at T1 was not significantly different between the two groups, but significantly lower HR (58 ± 12 versus 66 ± 11 beats·min^−1^; *P* < 0.001) and higher BIS (47 ± 5 versus 45 ± 3; *P* = 0.04) were evident in the H group at T1. MBP (74 ± 11 mmHg in H group versus 86 ± 19 mmHg in L group; *P* < 0.001), HR (61 ± 12 beats·min^−1^ in H group versus 70 ± 14 beats·min^−1^ in L group; *P* < 0.001), and BIS (48 ± 5 in H group versus 54 ± 5 in L group; *P* < 0.001) at T2 had significant differences between the two groups. The number of patients who needed vasopressors such as phenylephrine, ephedrine, and atropine and total doses of vasopressors were similar between two groups (Table 2). Vasodepressor like nicardipine and esmolol was not used in both groups during anaesthesia.

**Figure 2:**
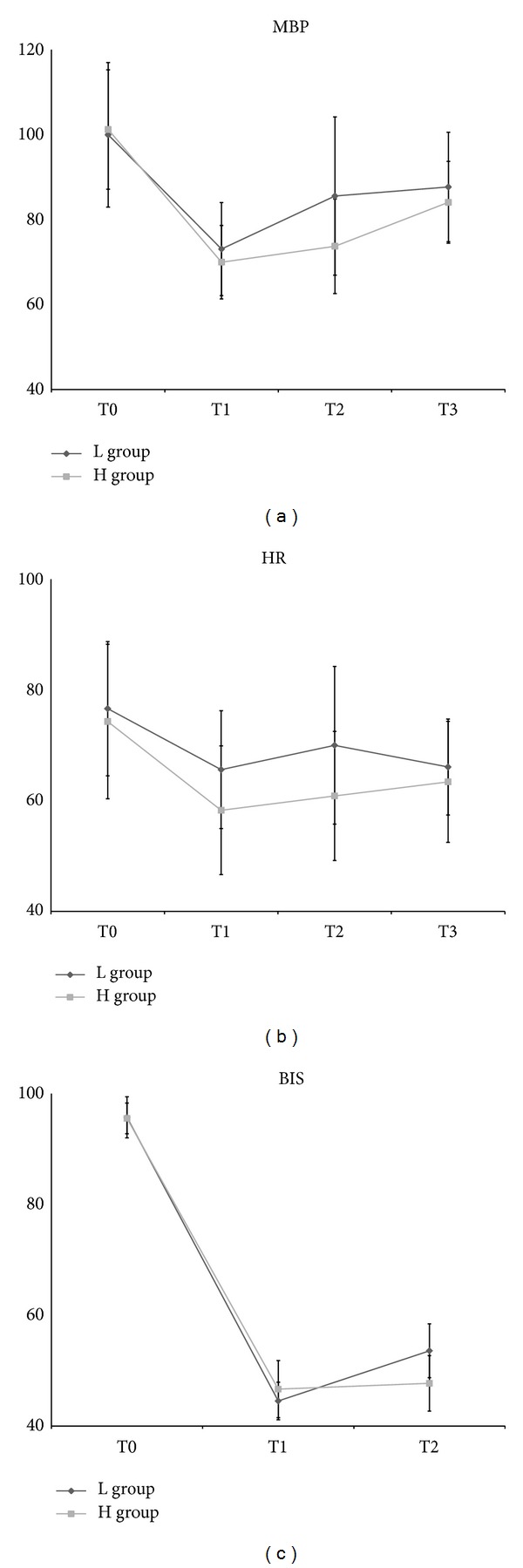
Haemodynamic changes according to time. (a) MBP: systemic mean blood pressure. (b) HR: heart rate. (c) BIS: bispectral index. L group: propofol-low dose remifentanil group; H group: propofol-high dose remifentanil group; T0: baseline value; T1: just before endotracheal intubation; T2: just after endotracheal intubation; T3: on arrival at postanesthetic care unit.

Concerning intragroup haemodynamic changes, MBP, HR, and BIS had no significant differences before and after endotracheal intubation (T1 and T2) in the H group, although significant differences were evident for MBP, HR, and BIS between T0 and T1 and between T0 and T2, respectively. MBP, except for HR and BIS, displayed significant differences before and after endotracheal intubation (T1 and T2) in the L group. Significant differences were evident in MBP, HR, and BIS between T0 and T1 and between T0 and T2, respectively (Figure 2).

**Figure 3:**
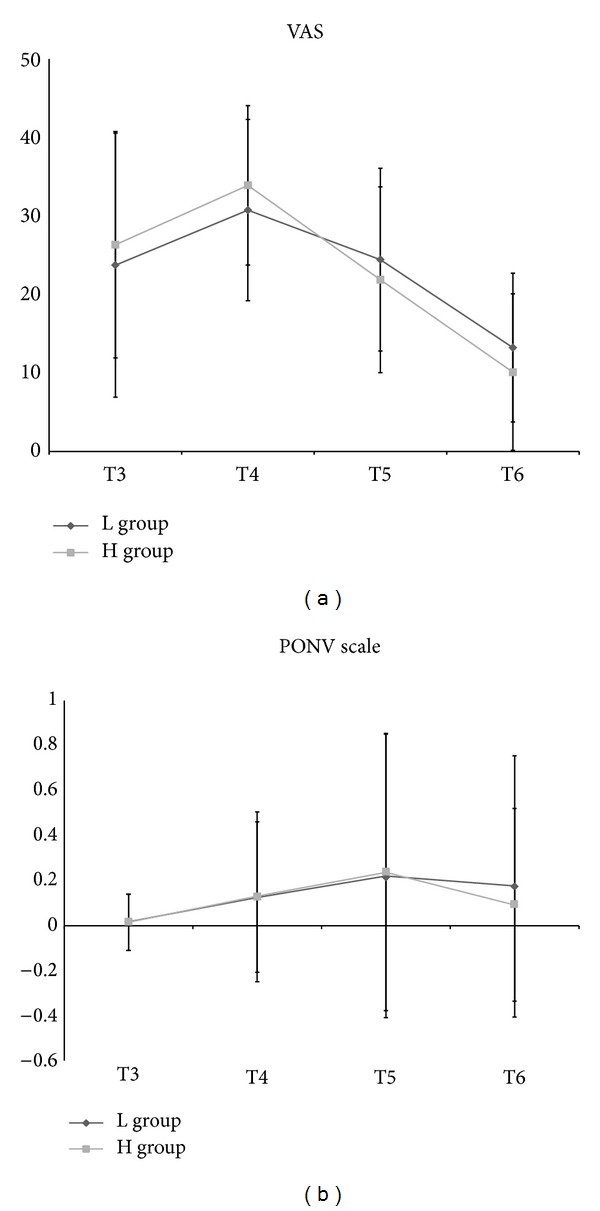
Postoperative pain and postoperative nausea and vomiting (PONV). (a) VAS: visual analogue scale. (b) PONV scale. L group: propofol-low dose remifentanil group; H group: propofol-high dose remifentanil group; T3: on arrival at postanesthetic care unit (PACU); T4: 30 min after arrival at PACU; T5: at 6 h after discharge from PACU; T6: at 24 h after discharge from PACU (T6).

**Figure 4:**
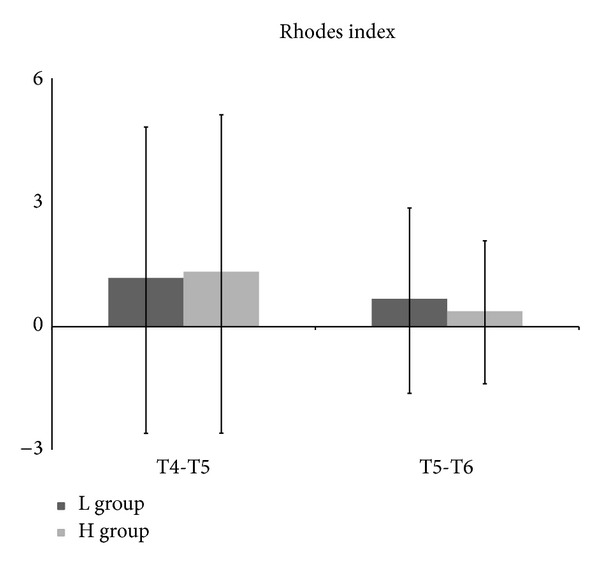
Severity of postoperative nausea and vomiting from 30 min after arrival at postanesthetic care unit (PACU) (T4) to 6 h after discharge from PACU (T5) and from 6 h after discharge from PACU (T5) to 24 h after discharge from PACU (T6) using Rhodes index. L group: propofol-low dose remifentanil group; H group: propofol-high dose remifentanil group.

Postoperative VAS and the number of analgesic treatments for postoperative analgesia on demand according to time had no significant difference between the two groups. The number of patients with PONV in the H group was one at T3, eight at T4, nine at T5, and seven at T6. The number of patients with PONV in the L group was one at T3, seven at T4, ten at T5, and four at T6. No significant difference of PONV incidence was evident. PONV scale, Rhodes index, and the number of antiemetic treatments with ondansetron on demand according to time were not significantly different between the two groups (Table 3, Figures 3 and 4).

## 4. Discussion

No difference of PONV between low- and high-dose of remifentanil in adult female patients with ASA PS classification I undergoing local excision of breast under TIVA was evident.

Propofol is an antiemetic agent, although the mechanisms are not clear [17, 18]. Fujii and Nakayama showed that a low dose of propofol (0.5 mg·kg^−1^) at the end of surgery is effective in preventing PONV during the first 24 h after anaesthesia in patients undergoing laparoscopic surgery [19]. Higher doses of propofol consumption were used in the present study (580 ± 294 mg in L group and 428 ± 138 mg in H group), including induction dose and dose for prevention of PONV. The PONV preventative concentrations of propofol in the blood or the effect-site have been unclear. However, continuous infusion of propofol with TCI might maintain the higher levels for longer time in the blood or the effect-site after the surgery, compared with two bolus doses of induction and small dose at the end of surgery, because the context-sensitive half time of propofol increases as the infusion time is lengthened [20]. Therefore, propofol combined with remifentanil in the present study might have prevented PONV in both groups although H group showed the lower consumption of propofol.

Several studies have an association of high-dose remifentanil with acute intolerance associated with high VAS score and the need for more analgesic agents [21, 22]. However, presently, postoperative VAS during the first 24 h after operation was not significantly different between the two groups. At first, the pain after local excision of breast was not severe in the L group and the injection of ketorolac at the end of the surgery might almost cover the postoperative pain with or without remifentanil-induced hyperalgesia, although some patients needed rescue ketorolac. Secondly, acute tolerance occurs with even a low-dose of remifentanil [23], but it has remained unclear whether the intensity of acute tolerance is dependent on the dose of remifentanil, or not. If remifentanil-induced hyperalgesia occurred in the present study, it would be observed in both groups, regardless of intensity. Therefore, the pain severity would not differ between two groups if the injected ketorolac relieved the postoperative pain. Thirdly, propofol delays and weakens the antianalgesic effect of remifentanil through various pathways [24–27]. Shin et al. reported that remifentanil-induced hyperalgesia was apparent with sevoflurane but not with propofol [28]. The total amounts of propofol were different between the two groups, but all patients in both groups were sedated with a continuous infusion of propofol. This might have led to the abolishment of the difference of pain severity between two groups in the present study. If an inhalational agent as a hypnotic agent was used in the present study, the result may well have been different.

One consideration remains. Gaszynski et al. did not explain the reason for the association of higher rate of PONV and postoperative pain with remifentanil bolus dose of 1 *μ*g·kg^−1^ for intubation and continuous infusion of 0.25–1.50 *μ*g·kg^−1^·min^−1^ in morbidly obese patients during open Roux-en-y gastric bypass [11]. Morbidly obese patients were not included in the present study. Although the higher concentration of remifentanil in the study of Gaszynski et al., compared with that in the present study, was associated with higher rates of PONV and postoperative pain, morbidly obese patients should have been included to generalize the results of the present study.

Bradycardia and hypotension are commonly encountered adverse effects of TIVA with remifentanil and propofol. The decrease of HR and MBP was also evident in the present study, regardless of the dose of remifentanil, with no similar total doses of phenylephrine, ephedrine, and atropine, although the extent of the decreases of HR and MBP was greater in the H group. However, the rise of HR and MBP after endotracheal intubation was observed only in the L group. The most important reason to use opioid in anaesthesia induction is to blunt sympathetic activations after endotracheal intubation and achieve haemodynamic stability. An adequate dose of opioid use without over- or under-dose is critical for the purpose. The H group showed more haemodynamic stability after endotracheal intubation than the L group, although no patient received nicardipine or esmolol to blunt sympathetic activations after endotracheal intubation in either group. Additionally, an intense stimulus, such as endotracheal intubation, significantly increased BIS in the L group indicating the possibility of intraoperative awareness at endotracheal intubation because of inadequate analgesia, although BIS before endotracheal intubation was in a tolerable general anaesthetic range between 40 and 60.

## 5. Conclusions

TIVA with high-dose remifentanil did not aggravate PONV with similar postoperative pain in adult female patients with ASA PS classification I undergoing local breast excision, compared with low-dose remifentanil. Furthermore, high-dose remifentanil showed more haemodynamic stability after endotracheal intubation than low-dose remifentanil.

## Figures and Tables

**Table 1:** Demographic data from the L and H groups.

	L group (*N* = 63)	H group (*N* = 63)	*P*
Age (years)	47 ± 12	47 ± 9	0.95
Height (cm)	158 ± 7	158 ± 5	0.91
Weight (kg)	57 ± 10	58 ± 8	0.55
Smoking (pack × years)	0 ± 1	1 ± 4	0.64
Hx of motion sickness	4	7	0.34
Hx of PONV	1	1	1.00
Remifentanil (*μ*g)	1013 ± 436	1894 ± 735	<0.001
Propofol (mg)	580 ± 294	428 ± 138	<0.001
Anaesthesia time (min)	104 ± 38	95 ± 35	0.15
Operation time (min)	69 ± 36	61 ± 33	0.23
Recovery time (min)	14 ± 6	13 ± 4	0.82

Values are expressed as number of patients or mean ± standard deviation.

L group: propofol-low dose remifentanil group; H group: propofol-high dose remifentanil group; Hx: history; PONV: postoperative nausea and vomiting.

**Table 2:** Haemodynamic parameters and bispectral index during anaesthesia.

	L group	H group	*P*
T0			
MBP (mmHg)	100 ± 17	101 ± 14	0.66
HR (beats·min^−1^)	77 ± 12	74 ± 14	0.17
BIS	96 ± 4	96 ± 3	0.18
T1			
MBP (mmHg)	73 ± 11	70 ± 9	0.13
HR (beats·min^−1^)	66 ± 11	58 ± 12	<0.001
BIS	45 ± 3	47 ± 5	0.04
T2			
MBP (mmHg)	86 ± 19	74 ± 11	<0.001
HR (beats·min^−1^)	70 ± 14	61 ± 12	<0.001
BIS	54 ± 5	48 ± 5	<0.001
T3			
MBP (mmHg)	88 ± 13	84 ± 10	0.09
HR (beats·min^−1^)	66 ± 9	63 ± 11	0.13
BIS	—	—	—
Vasopressor			
Phenylephrine			
Incidence	16/63	16/63	1.00
Dosage (*μ*g)	27 ± 101	31 ± 116	0.98
Ephedrine			
Incidence	2/63	2/63	1.00
Dosage (*μ*g)	0.13 ± 0.71	0.19 ± 0.86	0.88
Atropine			
Incidence	9/63	13/63	0.35
Dosage (*μ*g)	0.07 ± 0.18	0.10 ± 0.20	0.65
Vasodepressor			
Nicardipine (mg)	—	—	—
Esmolol (mg)	—	—	—

Values are expressed as mean ± standard deviation or number of patients.

L group: propofol-low dose remifentanil group; H group: propofol-high dose remifentanil group; T0: baseline value; T1: just before endotracheal intubation; T2: just after endotracheal intubation; T3: on arrival at postanesthetic care unit; MBP: mean systemic arterial blood pressure; HR: heart rate; BIS: bispectral index.

**Table 3:** Postoperative pain assessed by a visual analogue scale (VAS) and postoperative nausea and vomiting (PONV).

	L group	H group	*P*
T3			
VAS	24 ± 17	26 ± 14	0.37
PONV incidence	1	1	1.00
PONV scale	0.02 ± 0.13	0.06 ± 0.13	1.00
Analgesic	4	3	0.70
Antiemetic	0	0	1.00
Rhodes index	—	—	—
T4			
VAS	31 ± 12	34 ± 10	0.06
PONV incidence	8	7	0.90
PONV scale	0.13 ± 0.34	0.13 ± 0.38	0.90
Analgesic	5	5	1.00
Antiemetic	0	0	1.00
Rhodes index	—	—	—
T5			
VAS	24 ± 12	22 ± 12	0.22
PONV incidence	9	10	0.88
PONV scale	0.22 ± 0.63	0.24 ± 0.62	0.88
Analgesic	2	0	0.15
Antiemetic	0	1	0.32
Rhodes index	1.11 ± 3.72	1.25 ± 3.86	0.88
T6			
VAS	13 ± 9	10 ± 10	0.06
PONV incidence	7	4	0.64
PONV scale	0.18 ± 0.58	0.10 ± 0.43	0.64
Analgesic	0	0	1.00
Antiemetic	0	0	1.00
Rhodes index	0.64 ± 2.25	0.35 ± 1.74	0.63

Values are expressed as mean ± standard deviation or number of patients.

L group: propofol-low dose remifentanil group; H group: propofol-high dose remifentanil group; T3: on arrival at post-anesthetic care unit (PACU); T4: after 30 minutes on arrival at PACU; T5: at 6 hours after discharge from PACU; T6: at 24 hours after discharge from PACU; PONV scale: PONV assessed using a three-point ordinal scale (0 = none, 1 = nausea, 2 = retching, and 3 = vomiting).
