# Corrigendum to “Total Intravenous Anaesthesia with High-Dose Remifentanil Does Not Aggravate Postoperative Nausea and Vomiting and Pain, Compared with Low-Dose Remifentanil: A Double-Blind and Randomized Trial”

**DOI:** 10.1155/2019/5694912

**Published:** 2019-01-01

**Authors:** Seong-Hyop Kim, Chung-Sik Oh, Tae-Gyoon Yoon, Min Jeng Cho, Jung-Hyun Yang, Hye Ran Yi

**Affiliations:** ^1^Department of Anaesthesiology and Pain Medicine, Konkuk University Medical Center, Konkuk University School of Medicine, Seoul, Republic of Korea; ^2^Institute of Biomedical Science and Technology, Konkuk University School of Medicine, Seoul, Republic of Korea; ^3^Department of Surgery, Konkuk University Medical Center, Konkuk University School of Medicine, Seoul, Republic of Korea; ^4^Post-Anaesthetic Care Unit, Konkuk University Medical Center, Konkuk University School of Medicine, Seoul, Republic of Korea

In the article titled “Total Intravenous Anaesthesia with High-Dose Remifentanil Does Not Aggravate Postoperative Nausea and Vomiting and Pain, Compared with Low-Dose Remifentanil: A Double-Blind and Randomized Trial” [1], there was an error in the Results section, where “The number of patients with PONV in the H group was one at T3, eight at T4, nine at T5, and seven at T6. The number of patients with PONV in the L group was one at T3, seven at T4, ten at T5, and four at T6.” should be “The number of patients with PONV in the H group was one at T3, seven at T4, ten at T5, and four at T6. The number of patients with PONV in the L group was one at T3, eight at T4, nine at T5, and seven at T6.”
